# The development of a strategy for tackling health inequalities in the Netherlands

**DOI:** 10.1186/1475-9276-3-11

**Published:** 2004-10-23

**Authors:** Johan P Mackenbach, Karien Stronks

**Affiliations:** 1 Department of Public Health, Erasmus MC, University Medical Center Rotterdam, P.O. Box 1738, 3000 DR Rotterdam, the Netherlands; 2 Department of Social Medicine, Academic Medical Centre, University of Amsterdam, Meibergdreef 15, 1105 AZ Amsterdam, the Netherlands

## Abstract

Over the past decade, the Dutch government has pursued a research-based approach to tackle socioeconomic inequalities in health. We report on the most recent phase in this approach: the development of a strategy to reduce health inequalities in the Netherlands by an independent committee. In addition, we will reflect on the way the report of this committee has influenced health policy and practice.

A 6-year research and development program was conducted which covered a number of different policy options and consisted of 12 intervention studies. The study results were discussed with experts and policy makers. A government advisory committee developed a comprehensive strategy that intends to reduce socioeconomic inequalities in disability-free life expectancy by 25% in 2020. The strategy covers 4 different entry-points for reducing socioeconomic inequalities in health, contains 26 specific recommendations, and includes 11 quantitative policy targets. Further research and development efforts are also recommended.

Although the Dutch approach has been influenced by similar efforts in other European countries, particularly the United Kingdom and Sweden, it is unique in terms of its emphasis on building a systematic evidence-base for interventions and policies to reduce health inequalities. Both researchers and policy-makers were involved in the process, and there are clear indications that some of the recommendations are being adopted by health policy-makers and health care practice, although more so at the local than at the national level.

## Introduction

Before 1980, socioeconomic inequalities in health were a non-issue in public health (research) in the Netherlands. This changed in the early 1980's as a result of the publication of the Black Report in England [1], and a report on inequalities in health between neighborhoods in the city of Amsterdam [2]. Gradually, interest in health inequalities rose, first among researchers and then among policy-makers. Interest among policy-makers was further strengthened by the "Health For All by the year 2000" targets of the World Health Organization that the Dutch government officially endorsed in 1985 [3]. In 1986, the Ministry of Health published its Health 2000 report which was the first government document to include a paragraph on socioeconomic inequalities in health [4]. This was followed in 1987 by a conference organized by the prestigious Scientific Council for Government Policy, the outcome of which was a recommendation to start a research program on health inequalities [5] (see Table 1).

**Table 1:** Summary of policy developments from 1980 to 2000

1985	The Dutch government adopted the WHO Health For All policy targets
1986	Publication of the Health 2000 Report [15] by the Ministry of Welfare, Health and Cultural Affairs, including a paragraph on socioeconomic inequalities in health
1987	National conference on socioeconomic inequalities in health, organized under the aegis of the Scientific Council for Government Policy, resulting in a proposal for a national research programme (1989–1993) funded by the ministry of Welfare, Health and Cultural Affairs
1991	National conference, again organized under the aegis of the Scientific Council for Government Policy, resulting in an agreement among several parties involved to implement activities to reduce inequalities in health
1994	Results of the first national research programme were reported to the Minister of Public Health
1995	Publication of an important policy document by the Ministry of Public Health, Welfare and Sport (*Health and Wellbeing*). Reduction of socioeconomic inequalities in health was mentioned as one of the policy goals. Initiation of second national research programme (1995–2000)
1996	Publication of a second document on *Public Health Status and Forecasts*, by the National Institute of Public Health and Environmental Protection. Socioeconomic inequalities in health were stressed as a major public health problem
2000	Report of the Lemstra committee on the enforcement of public health. The reduction of socioeconomic inequalities was mentioned as an important policy aim.
	Growing demand by the Ministry of Public Health and parliament for information on effective interventions to reduce inequalities in health
2001	Results of the second national research programme, and recommendations based on these results, reported to the Minister of Public Health

Since then, the Dutch Ministry of Health has followed a systematic, research-based approach to tackling socioeconomic inequalities in health. An initial five-year research program mapped the nature and determinants of socioeconomic inequalities in health in the Netherlands [6]. A second six-year program launched in 1994 sought to gain systematic experience with interventions and policies designed to reduce socioeconomic inequalities in health. We report on the final phase of the second program: the development of a strategy to tackle health inequalities, and the production of a report containing recommendations for health policy making [7]. These recommendations were partly based on the results of the evaluation studies included in the second program. In addition, we will reflect on the way this report has influenced health policy and practice.

The report deals with socioeconomic inequalities in health, defined as systematic differences in health status between people with higher and lower socioeconomic status, as indicated by educational level, occupational class, and/or income level. Like other European countries, the Netherlands has substantial inequalities in health between socioeconomic groups. Differences in life expectancy at birth between socioeconomic groups are in the order of 4 years, and differences in healthy life expectancy have recently been calculated to be a staggering 14 years [8]. Inequalities in health care utilization, on the other hand, are quite modest, not only in an absolute sense [9], but also in comparison with other European countries [10]. In addition to socioeconomic health inequalities there are other important variations in health as well, e.g. between genders, regions, ethnic groups, and other socio-demographic variables [11]. Some of these are interwoven with socioeconomic inequalities in health, but the two programs mentioned above have tried to separate out the socioeconomic dimension from the other dimensions, in order not to dilute attention across too wide an area.

## Case study

### The research and development program

The main focus of the program was on developing and evaluating interventions and policies, but a number of other activities (monitoring of health inequalities, longitudinal explanatory study, research seminars, publications, documentation centre) were undertaken as well. Table 2 lists the evaluation studies that were commissioned after two calls for proposals and assessment by peer review. All interventions were aimed at tackling well-known determinants of socio-economic inequalities in health, such as poverty, smoking, working conditions, and accessibility of health care. Evaluation studies started between 1997 and 1999. The majority had a quasi-experimental design and compared health outcomes (e.g. school absenteeism) or intermediate measures (e.g. folic acid use) between an experimental and a control group. Positive results were reported for seven interventions: integrated program to prevent school children to start smoking, teeth brushing at primary school, adapted working methods and equipment for brick-layers, rotation of tasks among dustmen, formation of local care networks, peer education for Turkish diabetics, and introduction of nurse practitioners for asthma/Chronic Obstructive Pulmonary Disease patients. The other evaluation studies either failed because of an inadequate evaluation design or produced negative results [12]. [see Table 2].

**Table 2:** Intervention studies undertaken within the second national program on socioeconomic inequalities in health

*Interventions targeting socioeconomic disadvantage*
• Supplementary benefits to parents living in poverty, identified during preventive health screening of children (no evidence on effectiveness collected)
*Interventions targeting health-related selection*
• Counselling of secondary school children with frequent school absence due to illness (evaluation design failed)
*Interventions targeting factors mediating the effect of socioeconomic disadvantage on health*
• Tailored mass media campaign to promote periconceptional folic acid use (intervention did not reduce socioeconomic gap in folic acid use)
• Community-based intervention to improve health-related behavior in deprived neighborhoods (evaluation results will become available in 2002)
• Integrated program (including social skills teaching and monetary rewards) to prevent school children in lower general and vocational education to start smoking (intervention reduced smoking initiation rate)
• Teeth brushing at primary schools (intervention eliminated socioeconomic gap in teeth brushing)
• Adapted working methods (raised brick-laying) and equipment (lifting machine) for brick-layers (intervention reduced physical workload and sickness absenteeism)
• Rotation of tasks (driving and minicontainer loading) among dustmen (intervention reduced physical workload and sickness absenteeism)
• Introduction of self-organising teams in various production organisations (evaluation design failed)
*Interventions targeting accessibility and quality of health care services*
• Formation of local care networks among general practitioners, housing corporation staff and police officers to prevent homelessness among chronic psychiatric patients (intervention reduced house evictions and forced admissions to psychiatric hospitals)
• Peer education to diabetic patients of Turkish origin (intervention improved glycaemic control and healthy behaviour, but only in women)
• Introduction of nurse practitioners for asthma/COPD patients to general practice in deprived areas (intervention increased treatment compliance and reduced exacerbations)

When the results of the evaluation studies became available, meetings were held in 2000 with scientific experts and representatives from policy makers and from practice in six different areas (income, education, health promotion, working conditions, housing conditions, health care). During these meetings possible recommendations for new policies and interventions were tested and refined [13]. The input for the meetings not only included the results of the evaluation studies, but also added two additional papers. The first paper, drawn up by a scientist, gave an overview of effective interventions to reduce socioeconomic inequalities in health in that area. In the second paper, the implications of this overview for policy were analysed by an author with experience in that specific policy area (e.g. former secretary of state for educational affairs and the former minister of social affairs). The meetings contributed to a better understanding of current policy initiatives, and the major obstacles and promoting factors for a policy aimed at reducing inequalities in health.

### The government advisory committee

Subsequently, the committee overseeing the program held a number of plenary meetings to develop a comprehensive strategy to reduce health inequalities. Committee members were appointed by the Minister of Health, and they included former and active politicians of various political backgrounds, as well as a representative of the ministry of health and researchers. A conscious attempt was made to represent the whole (relatively narrow) political spectrum in the Netherlands. Members ranged from left (represented by the social-democrat mayor of the fourth largest city in the country) to right (represented by a former chairman of, and current House of Lords member for, the conservative party, who was later succeeded by another House of Lords member for the same party), and the committee was chaired by a former christian-democrat Minister of Social Affairs. Researchers had an important influence on the whole process: JM was secretary of the committee, and KS acted as co-ordinator of the program, and both were involved in writing draft versions of the final report. The committee reported directly to the Minister of Health.

### The rationale for the strategy

The committee started from the assumption that existing inequalities in health at least partly rank as unjust and that the government is responsible for achieving a reduction of these health differences. This assumption was based on the argument that health should be seen as a condition for the options open to individuals to structure their own life as far as possible according to their own ideas. Those health differences that are the consequence of an unequal distribution of living conditions over which individuals have no control, were thus seen as health inequities, to be tackled by the government. It was argued that this would require a comprehensive strategy, given the persistent and widespread character of socio-economic inequalities in health.

The committee wanted its strategy for reducing health inequalities to be based on sound evidence. Ideally, factors targeted by the strategy should be known to contribute to the explanation of health inequalities, and interventions and policies should be known to diminish exposure of lower socioeconomic groups to these factors. While the first requirement could be met relatively easily (and documentation was provided, with references, in the final report of the committee), the second requirement was more difficult to meet. Although the program produced evidence on effectiveness of interventions and policies and showed some positive results, this left important gaps in the knowledge base, both in terms of coverage of various policy options and in terms of strength of evidence. This problem was also encountered in other countries [14]. The committee considered that one cannot expect further evidence to become available unless large-scale measures to reduce inequalities in health are taken. It therefore decided to recommend a combination of implementation of 'promising' interventions with continued evaluation efforts. For each of the interventions and policies that were recommended for implementation, it carefully listed the available evidence, plus references.

In addition, the committee also paid attention to the political feasibility of possible policy recommendations. This aspect was discussed during the plenary meetings, in the light of the (political) experience of the committee members as well as the outcome of the working conferences that were mentioned before.

### Targets

The committee decided to base its strategy on a number of quantitative targets, because these can aid in plotting a clear policy course and can function as milestones for interim assessments of the strategy. It took the World Health Organization target as its starting point [15], and reformulated it for the Netherlands as: "By the year 2020, the difference in healthy life expectancy between people with a low and people with a high socioeconomic status should be reduced from 12 to 9 years, due to a (stronger) increase in healthy life expectancy in the lowest socioeconomic groups."

In order to attain such an ambitious goal, major efforts are required, if only because during the last decades inequalities in health in the Netherlands have increased rather than decreased [16]. Although it was considered unwise to give up on the ambition laid down in this 'inspirational' target, the strategy focused on a set of 'intermediate' targets that seem feasible today or in the near future. These targets were chosen to represent each of the main entry-points for reducing socioeconomic inequalities in health, and were limited to intermediate outcomes for which quantitative data for the Netherlands are currently available.

### Package of policies and interventions

Table 3 lists the interventions and policies constituting the strategy recommended by the committee. The strategy covers all four entry-points and spans the entire range between 'upstream' measures targeting socioeconomic disadvantage and 'downstream' measures targeting accessibility and quality of health care services. Where current policies were expected to contribute to reducing health inequalities (education policies, income policies, work disability benefit schemes, health care financing schemes), the committee explicitly recommended continuation. This is by no means trivial, because none of these achievements of the past can be considered safe for the future. For example, the Dutch government is considering a reform of the health care financing system that could lead to reduced coverage of health care for those insured under the current public scheme, and then would jeopardize equal financial accessibility.

**Table 3:** Recommended interventions and policy measures

*Interventions and policies targeting socioeconomic disadvantage*
• Continuation of policies that promote educational achievement of children from lower socioeconomic families.
• Prevention of an increase of income inequalities through adequate tax and social security policies.
• Intensification of anti-poverty policies, particularly policies that relieve long-term poverty through special benefit schemes and assistance with finding paid employment.
• Further development and implementation of special benefit schemes for families whose financial situation threatens the health of their children.
*Interventions and policies targeting health-related selection*
• Maintaining benefit levels for long-term work disability, particularly for those who are fully work disabled and those who are partly work disabled due to occupational health problems
• Adaptation of working conditions for the chronically ill and disabled in order to increase their work participation.
• Health interventions among long-term recipients of social assistance benefits in order to remove barriers for finding paid employment.
• Further development and implementation of counselling schemes for school pupils with regular or long-term absenteeism because of health problems.
*Interventions and policies targeting factors mediating the effect of socioeconomic disadvantage on health*
• Adapting health promotion programs to the needs of lower socioeconomic groups, particularly by focusing on environmental measures including the introduction of free fruit at primary schools and an increase of the excise tax on tobacco.
• Implementation of school health promotion programs that target health-related behaviour (particularly smoking) among children from lower socioeconomic families.
• Introduction of health promotion efforts into urban regeneration programs.
• Implementation of technical and organisational measures to reduce physical workload in low-level occupations.
*Interventions and policies targeting accessibility and quality of health care services*
• Maintaining good financial accessibility of health care for people from lower socioeconomic groups
• Relieving the shortage of general practitioners in disadvantaged areas.
• Reinforcing primary health care in disadvantaged areas by employing more practice assistants, nurse practitioners and peer educators, e.g. for implementing cardiovascular disease prevention programs and better care for chronically ill persons.
• Implementation of local care networks aiming for the prevention of homeliness and other social problems among chronic psychiatric patients.

In a number of other areas, the committee recommended intensified or new policies. These recommendations were partly based on reported positive results of intervention studies. This applies to the recommendations relating to school health promotion programs, technical and organizational measures to reduce physical workload, reinforcement of primary care in disadvantaged areas by employing practice nurses and peer educators, and local care networks to prevent social problems among chronic psychiatric patients. The results of some of the other intervention studies led to recommendations for further development of those interventions, as in the case of special benefit schemes for families living in poverty and counseling schemes for school absenteeism. Most of the other recommendations, however, are primarily based on an understanding of the factors that have been shown to contribute to health inequalities, and of the best way to deliver interventions targeting these factors.

The committee did not attempt to estimate the costs of the recommended interventions and policies.

### Implementation

As experience has taught that implementing effective interventions should not be taken for granted, the committee advised that a steering group be formed to drive and control the process of implementing effective interventions. On the one hand, this should function as a highly visible focal point at which the expertise available in the Netherlands is made accessible to all relevant policy areas. On the other hand, the steering group should be able to act on its own initiative to capture and retain attention for socio-economic inequalities in health and to promote the implementation of policy proposals. Given these two functions, the committee advised including experts as well as representatives from the main relevant policy areas in the steering group.

### Research and development

Given the fact that research has not yet fully disclosed the origins of socioeconomic inequalities in health, the committee considered continuation of explanatory research to be vital because it may lead to new entry-points for intervention. The same applies to further development of effective interventions and policies. The committee therefore recommended evaluation of all recommended interventions and policies during and after their implementation.

### Presentation of the report

The committee published its main report in March 2001 [7]. The report was launched at a press conference, and presented to both the minister of health and the minister of the 'Major Cities policy'. It received wide media coverage. All major newspapers wrote extensively about the findings and recommendations, and these were also presented and discussed in various national television and radio programmes. Some criticism was heard as well. These include the argument that any (shared) responsibility on the part of the government for reducing socio-economic inequalities in health is at odds with the social trend towards stimulating individuals to take responsibility for themselves. This was discussed in the context of health related behaviour (smoking, nutritional pattern etc.) in particular.

A closing conference took place in October 2001. During that conference, the results of the evaluation studies as well as the proposed policy strategy were presented to a broad public, and reflected upon by, among others, Sir Donald Acheson from the UK. In addition, policy implications were discussed. Participants included researchers, policy makers and representatives from practice, not only from the public health and health care field, but also from other policy areas (social security, working conditions etc.).

### Follow-up

The official cabinet reaction to the recommendations presented to parliament in November 2001 was positive but further elaboration of the recommendations as well as decision-making was deferred to the next cabinet [17]. A new cabinet was formed after turbulent elections in spring 2002 but fell within 3 months, and did not make decisions on a strategy to reduce socioeconomic inequalities in health. New elections were held in January 2003.

The delay in political decision making does not seem to have hindered the implementation of specific interventions that were evaluated within the programme. So far, at least a few of the interventions that have been proven to be effective have been implemented on a larger scale. These include the integrated programme to prevent school children from starting smoking, and the local care networks for chronic psychiatric patients.

## Discussion

While many countries, including the UK, Sweden and Finland have had national research efforts in the field of socioeconomic inequalities in health during the second half of the 1990's, the Dutch program is unique for its emphasis on evaluation of interventions. More generally, the main distinguishing feature of the Dutch approach is its focus on commissioning evaluations of interventions. Although this was done in a systematic way, using an explicit conceptual and methodological framework, the program also had its obvious limitations. It had a modest budget (totalling 3 million Euro over a period of 6 years) and funded not more than 12, rather small-scale intervention studies targeting relatively easily modifiable factors. The latter is not only due to the small budget of the program, but also to strict methodological requirements which in practice made it nearly impossible to study the effectiveness of broader policy measures [18]. In hind-sight, we consider this the most important limitation of the program: the lack of studies on the possible impact of broader policy measures, mainly related to the strict methodological criteria that were applied in the process of selection of the research proposals. Even for the more specific and narrowly defined interventions selected for the program, some of the evaluation studies failed because the design could not be implemented. In the end, therefore, the contribution of the intervention studies to strategy development was modest.

The unique elements of the Dutch approach should not distract from the fact that the Dutch experience received important inputs from abroad. Its start is a late response to the British Black Report and is directly related to the efforts of the European Office of the World Health Organization to put health equity on national policy agendas [15]. During the program there were close contacts between members of the committee and researchers and policy-makers in other European countries, through the European Network for Interventions and Policies to Reduce Inequalities in Health [19], so that experiences in other countries could be taken into account. The report of the Independent Inquiry in Britain [20] acted as a rich source of ideas, while a recent Swedish report on tackling inequalities in health [21] strengthened the confidence in the usefulness of target setting for reducing inequalities in health.

The Dutch approach reflects the input of both researchers and policy-makers, although the balance between the two has oscillated over time. The first signals that health inequalities should be addressed came from researchers, but were picked up by policy-makers within the Ministry of Health in the mid-1980's who were then looking for opportunities to strengthen health policy (as opposed to health care policy) in the Netherlands. This small group of bureaucrats succeeded in launching and following through the first research program, but left the Ministry or changed posts before the program came to an end. Partly due to continuous personnel changes in the Ministry, the intensity of the exchanges between researchers and policy-makers gradually diminished during the second program. When the final report was published reactions from within the Ministry were rather cool, although the Minister, who had taken a personal interest in the matter, responded very favourably. At this stage, however, it seems that without a continuing "push" from the research-side the bureaucrats could easily loose interest altogether, particularly now that there are rapid changes of cabinet.

A major obstacle for a comprehensive package of policy measures seems to be the relatively weak position of the Ministry of Health as compared to other policy areas. It is obvious that a substantial reduction of health inequalities can be achieved only by involving other policy areas next to that of (preventive and curative) health care. This starting point seems to contrast with the ideas of the ministries in other policy areas, that seem to consider this issue as the responsibility of the Ministry of Health in particular. So far, the Ministry of Health does not seem to have a lot of success in convincing other policy areas of the importance of contributing to reducing inequalities in health.

The lack of success in mobilising other policy areas at the national level is probably partly related to the fact that the issue of inequalities is perceived as rather abstract by these other areas. This probably requires the issue of inequalities in health to be "re-phrased" for that specific policy area, in terms that fit within their ideas. Housing corporations for example do not consider themselves to be responsible for tackling health inequalities but they do feel responsibility for high quality living conditions, which then might automatically contribute to a better health status of people in lower socio-economic groups. Paradoxically, an approach in which the issue of inequalities in health is cut into small pieces, requires a steering. This forms the background of the plea of the committee for a steering group.

Remarkable progress has been made, not only in terms of knowledge production but also in terms of increased confidence among policy-makers and practitioners to take action to reduce inequalities in health. Many health agencies in the Netherlands are working to reduce socioeconomic inequalities in health. This is illustrated by the fact that the 'National Contract on Public Health', concluded in 2001 between many national and local agencies in the field of public health, has selected the reduction of socioeconomic inequalities in health as its first priority. Many local health agencies have already implemented some of the interventions discussed in this paper.
